# Emerging Materials to Prepare Mixed Matrix Membranes for Pollutant Removal in Water

**DOI:** 10.3390/membranes11070508

**Published:** 2021-07-05

**Authors:** Yu Jie Lim, So Min Lee, Rong Wang, Jaewoo Lee

**Affiliations:** 1 Singapore Membrane Technology Center, Nanyang Environment and Water Research Institute, Nanyang Technological University, Singapore 637141, Singapore; yujie001@e.ntu.edu.sg; 2 School of Civil and Environmental Engineering, Nanyang Technological University, Singapore 639798, Singapore; 3 Interdisciplinary Graduate Programme, Graduate College, Nanyang Technological University, Singapore 637553, Singapore; 4 Department of Bionanotechnology and Bioconvergence Engineering, Jeonbuk National University, 567 Baekje-daero, Deokjin-gu, Jeonju-si 54896, Korea; sominlee@jbnu.ac.kr; 5 Department of Polymer-Nano Science and Technology, Jeonbuk National University, 567 Baekje-daero, Deokjin-gu, Jeonju-si 54896, Korea

**Keywords:** mixed matrix membranes, adsorbents, filler materials, nanomaterials, biomass, pollutant removal, ion exchange process, heavy metals, dye molecules, microplastics

## Abstract

Various pollutants of different sizes are directly (e.g., water-borne diseases) and indirectly (e.g., accumulation via trophic transfer) threatening our water health and safety. To cope with this matter, multifaceted approaches are required for advanced wastewater treatment more efficiently. Wastewater treatment using mixed matrix membranes (MMMs) could provide an excellent alternative since it could play two roles in pollutant removal by covering adsorption and size exclusion of water contaminants simultaneously. This paper provides an overview of the research progresses and trends on the emerging materials used to prepare MMMs for pollutant removal from water in the recent five years. The transition of the research trend was investigated, and the most preferred materials to prepare MMMs were weighed up based on the research trend. Various application examples where each emerging material was used have been introduced along with specific mechanisms underlying how the better performance was realized. Lastly, the perspective section addresses how to further improve the removal efficiency of pollutants in an aqueous phase, where we could find a niche to spot new materials to develop environmentally friendly MMMs, and where we could further apply MMMs.

## 1. Introduction

Water contamination by organic and inorganic pollutants emitted from anthropogenic activities is a serious global problem found everywhere regardless of whether the country is developed or developing. In most developing countries, wastewater treatment plants consist only of physical (primary) and biological (secondary) treatments due to the lack of proper infrastructure for tertiary treatment in many cases [1]. For that reason, 80% of municipal and industrial wastewater is discharged into the environment without adequate treatment in developing countries, according to the United Nations [2]. The polluted water that did not meet the discharge standard is likely to deteriorate the water quality and sanitary conditions, thereby increasing the risk of exposure to water-borne diseases [3]. When it comes to the developed countries, they have adequate facilities to provide fresh water to more than 90% of the population [4]. However, they also suffer from a variety of anthropogenic contaminants such as emerging biological (e.g., super bacteria, viruses) and chemical (hormones, endocrine, pharmaceuticals, toxins) micropollutants or industrial wastewater containing heavy metals and hard-to-manage acidic wastewater [5,6,7].

Membrane-based technologies used for water treatment can be an excellent alternative to handle wastewater including both conventional and emerging pollutants as they can provide technological (e.g., high quality of treated water) and economic (e.g., small footprint and low unit cost of production) advantages over other water treatment processes [8,9,10]. However, they still need to be further improved to ensure the relative price competitiveness and practical feasibility for highly efficient wastewater treatment, given that wastewater treatment is likely to require a much lower cost of treatment as compared to the production of drinking water. Accordingly, it is imperative to develop novel membranes that can meet the availability requirement with high throughput capability to realize practical applications for advanced wastewater treatment capable of removing diverse anthropogenic pollutants.

As a promising alternative, nanomaterial-based membranes can be effective, as nanomaterials are highly likely to offer distinct advantages over conventional polymeric materials used for membrane fabrication. Particularly, two-dimensional (2D) nanomaterials such as graphene oxide (GO) and Mxenes have shown great potentials when used in the form of laminate membranes in the filtration processes. In detail, they feature ultra-fast transport of water molecules, precise molecular sieving, and tunable physicochemical properties [11,12], and these characteristics make the laminate membranes possess superior filtration performance. However, the laminate membranes stemming from 2D nanomaterials are not without their problems. We cannot ignore the fact that those kinds of laminate membranes have serious limitations in terms of adverse environmental effects, safety issues, mass production, and scalable membrane area.

Specifically, 2D nanomaterials typically require a considerable amount of acid or hard-to-handle chemicals (e.g., H_2_SO_4_/HNO_3_ for GO [13]; fluorine-based etching agents for MXenes [12]). A volume of hard-to-handle wastewater generated from the manufacturing process of laminate membranes makes the laminate membranes difficult to be readily available for water treatment due to the inherent environmental load and safety issues. Moreover, the two issues hold back the mass production of laminate membranes. Lastly, to the best of our knowledge, it is almost impossible to fabricate large-area laminate membranes to the extent that they can be used for real applications. To address the limitations, a few research groups have made significant advancements in minimizing the consumption of toxic chemicals in the manufacturing process [14,15] or realizing a larger laminate membrane (13 × 14 cm^2^ [16]). However, it is generally true that a laminate membrane still has other fundamental limitations that need to be addressed before reaching the technological maturity level to meet the availability requirement for real applications.

In this regard, mixed matrix membranes (MMMs) comprising polymer matrix-containing fillers can be a viable alternative to overcome the above-mentioned limitations of laminate membranes imposed by the inherent properties of 2D nanomaterials. MMMs can confer additional functions (e.g., antifouling properties [17], enzyme immobilization [9], mechanical reinforcement [18], and adsorption for pollutant removal [19]) to a polymeric membrane with a minimum amount of nanomaterials for a specific purpose in the light of its concept (i.e., a membrane consisting of polymer matrix including fillers). Accordingly, MMMs are likely to be practically utilized for pollutant removal without the contradiction that the manufacturing process of membranes used for wastewater treatment paradoxically generates a large amount of hard-to-manage wastewater, thanks to the following advantages.

First, as aforementioned, only a small amount of nanomaterials is required to prepare MMMs compared to the total weight, thereby minimizing the environmental and safety issues accompanied by the preparation of nanomaterials. Second, MMMs can be readily reused by adjusting pH [20], contributing to the further reduction of hard-to-manage wastewater and plastic waste by decreasing the usage of both nanomaterials and polymeric membranes. Third, MMMs can simultaneously remove different types of pollutants from an aqueous solution by both adsorption and size exclusion. Lastly, it is easier to commercialize MMMs than laminate membranes since MMMs are much more suitable for the mass production of large-area membranes applicable to standard membrane modules used in the wastewater treatment industry. With these points in mind, in this paper, we review the research progresses and trends on the emerging materials used to prepare MMMs for pollutant removal from water streams in the recent five years.

## 2. Literature Analysis on Mixed Matrix Membranes Used for Pollutant Removal

A literature survey to investigate the research trend related to MMMs for pollutant removal since 2010 was carried out based on the search using “mixed matrix membranes” and “pollutant removal” as specific keywords. The review papers, books, theses, and patents relevant to MMMs for pollutant removal were excluded from consideration. Among the searched research articles, nanocomposite adsorbents, two-dimensional nanomaterial-based laminates, and nanomaterial-coated membranes were excluded based on the definition of MMM (i.e., a membrane consisting of polymer matrix including fillers). In addition, MMMs for gas separation were also excluded, given that this paper focuses on pollutant removal in water. 

**Figure 1:**
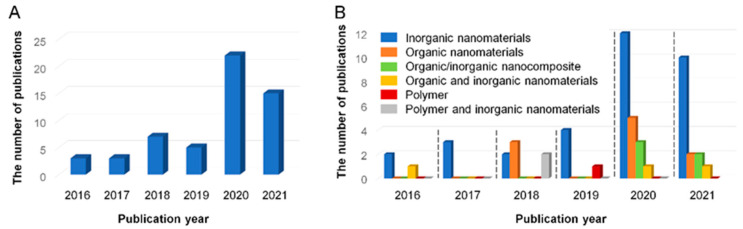
(**A**) The number of publications of research papers related to MMMs for pollutant removal. (**B**) The number of publications of research papers related to MMMs prepared with different types of additives for pollutant removal. The numbers of publications in 2021 in Figure 1A,B were obtained from the literature survey performed until the first quarter of 2021. The data in Figure 1 were obtained from the ScienceDirect database on 20 May 2021.

Although the literature survey was performed for the research papers published since 2010, a total of 55 papers were found to be published from 2016 to the first quarter of 2021. This result means that the topic related to MMMs for pollutant removal has only recently begun to be studied and is still at an early stage. A more important point is the increasing trend in the number of publications (Figure 1A). Three papers were published in the earliest two years (2016 and 2017), and the number of publications was doubled in the following two years (2018 and 2019). A big increment in the number of publications was seen in 2020, which is almost 3.7 times higher than that in 2019, and it reached almost 70% of 2020 in the first quarter of 2021. This tendency shows that there is a growing interest in MMMs for pollutant removal. 

Another point worthy of note is the most preferred additive type to prepare MMMs for pollutant removal. To be specific, inorganic nanomaterials have been most frequently used as an additive throughout the whole period, which was evidenced by Figure 1B. This observation could suggest that inorganic nanomaterials might be the most appropriate to endow polymeric membranes with a certain function pertaining to adsorption capacity or removal efficiency for pollutants in water when incorporated into a polymer matrix. The next best type of additives was organic nanomaterials. It seems that the organic/inorganic nanocomposite additives have begun to be used to prepare novel MMMs for pollutant removal since 2020.

## 3. Recent Progress in Pollutant Removal Using Mixed Matrix Membranes

MMMs are heterogeneous membranes prepared by incorporating inorganic fillers with high selectivity into a polymer matrix featuring excellent mechanical properties and processability. More broadly, an MMM can be defined as a membrane consisting of a polymer matrix containing “homogeneously dispersed fillers (not limited to inorganic fillers)” with different selectivity and transport properties as described in the literature [21]. Note that we refrain from using “single-phase materials” to describe MMMs since dispersed fillers (i.e., a dispersed medium) in a polymer matrix should be distinguished from a continuous medium (i.e., a polymer matrix) in a microscopic point of view since fillers are not dissolved but dispersed in a polymer matrix, inevitably leading to two-phase materials. To reap the benefits of the fillers in the MMM, it is crucial to properly disperse the former in the continuous matrix, which means that it is necessary to minimize filler agglomeration in liquid form prior to the fabrication process [22]. However, some filler materials tend to agglomerate (e.g., due to inherent hydrophobicity), and empirical evidence suggests that agglomeration is usually more pronounced under high filler or additive concentrations [23,24]. Hence, methods such as oxidation or modification of filler properties (to increase compatibility with the polymer matrix), ultrasonication as well as the addition of surfactants are typically employed to physically disperse the filler materials in the liquid phase prior to the membrane fabrication process [25,26,27].

**Figure 2:**
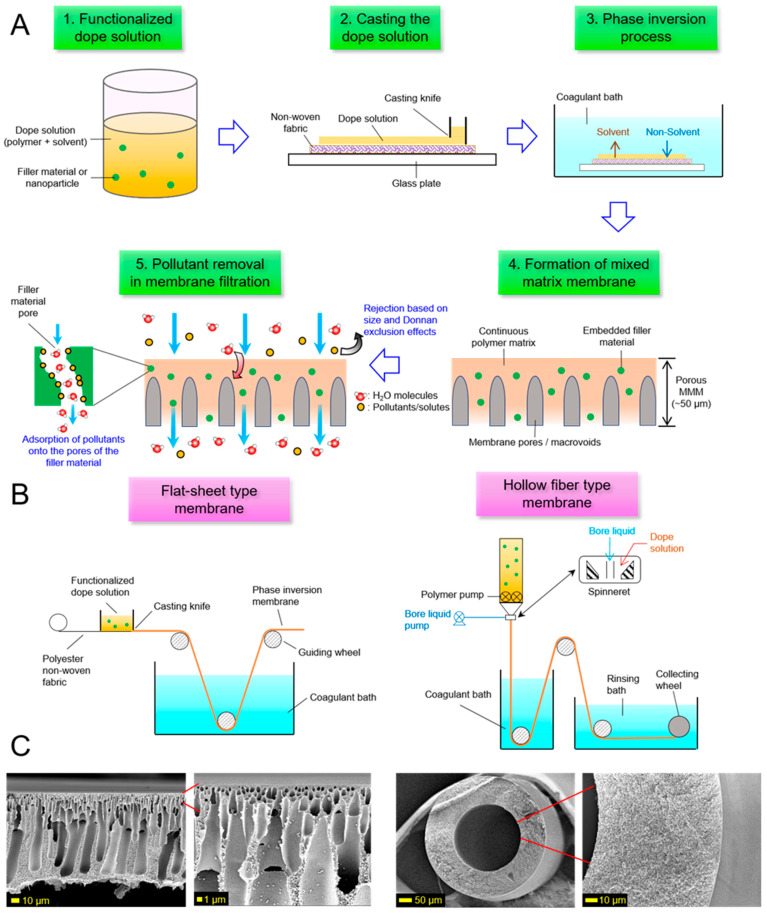
(**A**) Schematic illustration of the methodology to fabricate porous MMMs via the phase inversion process in a lab-based setting. The filler materials are embedded into the continuous polymer matrix. The pollutant rejection and removal mechanisms are the size exclusion and Donnan exclusion effect by the membrane as well as the adsorption of pollutant ions onto the inner pores of the filler material. (**B**) Schematic illustration of the large-scale production of flat sheet and hollow fiber type MMM via membrane casting (left) and spinning technique (right). (**C**) Scanning electron microscopy (SEM) images of porous membranes. The left-most two images outline a flat-sheet Polysulfone (PSf) membrane that is dominated by tiny macrovoids at the top and big macrovoids at the bottom. The right-most two images outline a hollow fiber Matrimid 5218 (Polyimide) membrane that has a fully sponge-like structure. All SEM images were taken at an operating voltage of 5.0 kV.

Getting back to the point, as an MMM can be broadly defined as above, a great variety of MMMs can be prepared by combining various kinds of polymers and fillers. Regardless of types of polymers and fillers, MMMs are typically prepared via a straightforward non-solvent induced phase separation (NIPS) method in most cases (Figure 2A,B) as common polymeric microfiltration or ultrafiltration membranes (Figure 2C). This point implies that various types of novel MMMs can be fabricated and scaled up using the existing production lines and infrastructure without the need to reinvent a fabrication technique or protocol needed for producing MMMs. Meanwhile, it boils down to the choice of fillers when it comes to how to endow an MMM with specific functionalities, given that the physical and chemical properties of the former can affect the characteristics of the latter. This fact suggests the possibility that manufacturers can not only develop a wide range of MMMs but also make customized products readily accessible by using various kinds of fillers (e.g., to endow a membrane with a function specifically tailored for a particular application). These features of MMMs will offer the prospect of success in pragmatic trials in pollutant removal using MMMs. Indeed, a number of applications of MMMs prepared with several kinds of fillers have been implemented to remove heavy metals, dye molecules, humic acid, organic compounds, nitrates, ammonia, and so on [20,24,28,29,30,31,32,33,34,35]. Specific cases and detailed mechanisms underlying pollutant removal using MMMs are discussed throughout this section. 

### 3.1. Heavy Metal Removal

Heavy metals refer to chemical elements that have a high density (typically >5 g/cm^3^) and are typically toxic even at low concentrations [36]. Some prominent examples of heavy metals include arsenic (As), mercury (Hg), cadmium (Cd), and lead (Pb). Heavy metals are present in the environment because of natural and anthropogenic sources. The former includes weathering of rocks, whereas the latter includes industrial activities that produce wastewater such as textile and paint production or electronics manufacturing activities [37]. Heavy metals are known to be highly soluble in water and tend to be absorbed by living organisms. The pollution and contamination of aquatic systems due to the presence of heavy metals are a cause for concern because of their harmful effects as well as the non-biodegradability, which will eventually accumulate onto living organisms and lead to detrimental health effects such as skin cancer, cardiovascular diseases and brain damage [38,39]. To minimize the detrimental impacts brought about by heavy metals, the World Health Organization has imposed strict standards for the concentration of heavy metals in drinking water (e.g., <10 ppb of arsenic) [39]. Unlike organic pollutants that can be degraded naturally or through treatment by advanced oxidation processes (AOPs), heavy metals cannot be removed and is thus persistent in the environment if they are not physically removed [38].

Membrane-based technologies have been adopted to remove heavy metals from aqueous solutions because of their ease of operation, decent removal efficiencies, and most importantly, modest energy consumption [27,37]. For example, reverse osmosis (RO) membranes are known to attain high rejections (~95%) of heavy metals [39]. This is attributed to the dense, highly cross-linked nature of the selective layer with small pore sizes of ~0.25–0.8 nm [26,40], thereby severely restricting the passage of dissolved heavy metals. However, the energy consumption of RO is high, and thus it is not favorable to use dense RO membranes for heavy metal removal. The more economical method is to use porous ultrafiltration (UF) membranes to remove heavy metals. However, the much bigger pore size of UF membranes (i.e., poor size-exclusion capacity) cannot attain high rejections (>90%) of heavy metals [41]. To strike a balance between economic feasibility (in terms of energy consumption) and removal efficiency, researchers have proposed the use of functionalized porous membranes to improve the rejection of heavy metals. The idea is to incorporate adsorbent filler materials into the membrane matrix to enhance the removal of dissolved heavy metals (Table 1). Furthermore, it was reported that the blending of inorganic materials into the membrane polymer matrix typically enhances the latter’s mechanical, structural and chemical properties that may be beneficial for long-term usage in wastewater treatment.

**Figure 3:**
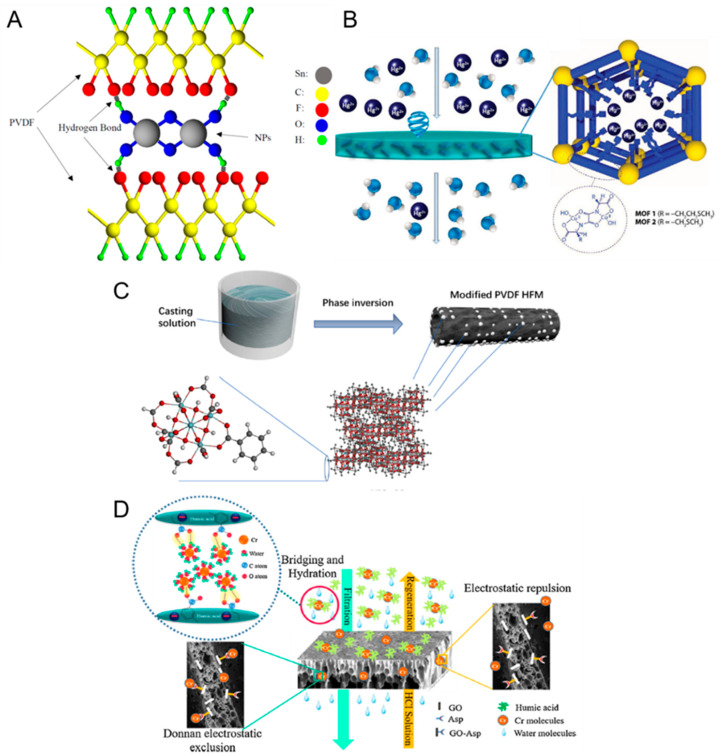
(**A**) Schematic illustration of the possible interaction between PVDF and SnO_2_ filler materials [37]. The filler materials are anchored onto the PVDF membrane matrix via hydrogen bonding. (**B**) Schematic illustration of the filtration process in which wastewater containing mercury ions is filtered through the MMM. The adsorption of mercury ions onto the MOFs occurs as a result of the interaction between mercury ions and functional groups in the interior of the MOFs channels [38]. (**C**) The fabrication procedure of a hollow fiber MMM incorporated with UiO-66 filler materials [39]. (**D**) The proposed mechanisms of filtration and regeneration processes of the MMM incorporated with aspartic acid-functionalized graphene oxide for chromium ion and humic acid removal [43]. The bulk membrane matrix is made of polyvinylchloride. The regeneration process is done via filtering the membrane with hydrochloric acid. All figures are reprinted with copyright permissions from the respective references.

Overall, the various methods to remove heavy metals using MMMs in Table 1 are based on two overarching mechanisms:

- ***Adsorption and electrostatic attractions.*** MMMs based on the incorporation of adsorbent materials into porous membranes have shown promise in the treatment of wastewater laden with heavy metals [27,37,42]. Typically, the adsorbent nanomaterial should have a high affinity with the heavy metal ions to enable the latter to be readily adsorbed by the MMM. Another important factor is that the nanomaterials should be stable (i.e., will not leach out) under a wide pH range (e.g., during chemical cleaning) to guarantee their long-term performance. Recently, Ibrahim et al. reported the incorporation of SnO_2_ filler materials into polyvinylidene fluoride (PVDF) membrane in a bid to increase the removal of various heavy metal ions [37]. One facile way to securely anchor the filler materials onto the membrane matrix is via hydrogen bonding [37,41] (e.g., SnO_2_ fillers in PVDF matrix, as shown in Figure 3A). Because of the inherent affinity of SnO_2_ with the heavy metal ions, the MMM was capable of removing heavy metals to a larger extent (up to a 20% increase in rejection) because of the enhanced adsorption. It was also postulated that the presence of inner-sphere complexes (that is, the direct bonding between the surface and the adsorbed species) led to the electrostatic attraction between SnO_2_ and metal ions which enhanced the adsorption of heavy metals onto the MMM [37]. Metal-organic frameworks (MOFs) have also shown promise as a filler material for pollutant removal in MMMs because of their intrinsic characteristics such as high metal binding capacities and a large surface area per unit volume (e.g., 918 m^2^/g for UiO-66 [39]) which enhances mass transfer in the sorption process (Figure 3B). The latter trait is particularly important because adsorption is deemed the most suitable removal method in treating feeds with a low concentration of heavy metals. For instance, Bruno et al. reported the success (>98% Hg^2+^ rejection) of MMMs incorporated with MOFs when removing trace amounts of mercury in the feed solution (2.61 ppm) [38]. The key message here is that the trait of large surface area per unit volume is typically desired when treating feeds with a low concentration of heavy metals to enhance the adsorption rate of the latter.

The adsorption rates might not occur uniformly throughout the filler material for that some functional groups of the material might have more favorable sites for adsorption or higher ion exchange capacities [37,39,43,46]. For example, it was revealed that the oxygen atom in the Zr-O-C bond of UiO-66 hollow fiber membranes (Figure 3C) was the most favorable site for arsenic sorption based on binding energy calculations [39]. It was hypothesized that the electrostatic attraction between UiO-66 and arsenate species, together with the adsorption of the latter onto the surface of UiO-66 accelerated the removal of arsenate [39]. Regression of the kinetics data also revealed that the adsorption process could involve more than one step. For example, it was reported that the sorption of Cr (VI) and Pb (II) ions occurs through two distinct steps before attaining equilibrium: (1) external mass transfer whereby metals ions are transferred onto the macro-pores of the filler in the MMM and (2) intra-particle diffusion between the mesopores of the filler in the MMM [22].

It is also worth noting that the pH of the feed is typically adjusted to maximize the adsorption capacity of the MMM for that the former controls the speciation of the heavy metal (e.g., as pH increases, H_3_AsO_4_ will be transformed into AsO_4_^3−^) as well as the charge of the membrane surface and heavy metal ions, which ultimately dictates the ion exchange capacity between the MMM and metal ions [39,41,42]. As another example, it was reported that the adsorption capacity of hexavalent chromium (Cr(VI)) was the highest under acidic conditions (pH = 2–3.5) because of its speciation whereby hydrogen chromate (HCrO_4_^−^) and dichromate (Cr_2_O_7_^2−^) are the dominant species [22]. It was postulated that the electrostatic attraction between the hexavalent anionic chromium and protonated hydroxyl and amino groups [22] or positively charged amine groups [48] led to the increased adsorption of Cr(VI) ions onto the MMM. In summary, the rejection of heavy metals using MMMs is typically contingent on the surface charge of the membrane, charge valency of the heavy metal as well as the ion concentration in the feed, all of which could be optimized to increase the rejection of the target heavy metal ion(s). 

The adsorption of heavy metals is known to follow the Langmuir (monolayer adsorption) or Freundlich (multi-layer adsorption) isotherms [39,44], both of which state that there exists a limit on the adsorption capacity with respect to the feed concentration. Thus, MMMs are usually less effective in treating feeds with a high concentration of heavy metals because adsorption sites will get saturated quickly. There is a need for a cleaning step to restore the membrane performance (i.e., restore the adsorption sites) once the active adsorption sites are saturated (usually evidenced by the drastic drop in removal efficiency of the MMM) [39,41]. Another approach is to engineer the adsorbent materials to be ‘regenerative’ (i.e., the active sites can be reinstated) by performing an in-situ acid backwashing process or using regeneration solutions under an electric field [43,44]. For example, it was postulated that the presence of H^+^ ions could replace the adsorbed Cr ions on graphene oxide (GO), thereby freeing up the adsorption sites (Figure 3D). In another study by Mukherjee et al. [47], it was reported that the adsorption sites in GO-hollow fiber membranes could be restored by the in-situ circulation of HCl solution within 30 mins. However, the study also noted that the rejection of hexavalent chromium (chromium (VI)) was reduced by ~7% after three consecutive cycles. It must be highlighted that the regeneration efficiency typically decreases with more cycles [44] because of incomplete desorption such that the initial adsorption sites cannot be fully regenerated. In addition, the drop in adsorption capacity of the MMM can also be attributed to the minor leaching of the adsorbent during the regeneration process [22]. Hence, to maintain the initial performance of the membrane, in the long run, the adsorbent material should be stable (i.e., will not leach out) and also able to retain its adsorption capacity after multiple cycles of filtration and regeneration.

- ***Size exclusion and Donnan exclusion effects.*** Besides adsorption, another facile yet promising method to increase the heavy metal removal efficiency of membranes is via size exclusion or Donnan exclusion effects. The latter has shown success by introducing functionalized adsorbents that are of opposite charge to the target metal ions [43]. Because the pores of a typical UF membrane cannot exclude heavy metals during filtration, researchers have attempted to circumvent this limitation by either modifying the size of the hydrated ions or the surface pore size of the UF membrane [27,41,43]. For example, the interaction between alumina particles and arsenic can lead to the formation of bigger complexes in size, thereby being more preferentially rejected by the pores of the UF membrane [27]. In another example, Namdar et al. reported a successful approach to simultaneously increase the rejection of chromium ions and humic acid via size exclusion effects [43]. It was hypothesized that the bridging of chromium ions with humic acid (via electrostatic attractions) led to the formation of bigger sized humic acid-chromium complexes which cannot permeate through the pores of the membrane so readily, thereby more readily being rejected by the membrane. This approach of size exclusion has also shown success in the fabrication of biopolymer-based MMM for heavy metal removal. As an example, it was reported that the MMM incorporated with CuO nanoparticles in the hydroxyethyl cellulose (HEC) biopolymer could attain high rejection of Cr(VI) and Pb(II) ions (hydrated radii of 0.38 nm and 0.4 nm, respectively) despite the much bigger pore size (3 nm) of the membrane [50]. It was postulated the presence of the hydration shell in the aqueous phase led to much bigger sizes of both ions, thereby readily rejected by the MMM via size exclusion.

In summary, the filler materials should have high porosity to increase the specific surface area available for heavy metal adsorption (e.g., 261 m^2^/g for alumina particles [27,42]). Also, the filler materials should be small and uniformly sized (e.g., 170 nm for zeolites [45] and 50 nm for alumina [27]) to maximize the surface area for adsorption or the binding with other ions (e.g., increasing the size of the hydrated ions so that they can be rejected by the membrane pores). However, the hygroscopic nature of some filler materials (e.g., Al_2_O_3_ nanoparticles) can lead to agglomeration, which is undesirable because it minimizes the surface area for adsorption. Hence, it is essential to modify them (e.g., zinc doping of Al_2_O_3_ particles [27]) to reduce the agglomeration propensity in a bid to maximize the MMM’s productivity and surface area available for adsorption.

### 3.2. Dye Removal

Dyes are colored substances that establish chemical bonds to the substrates (which they are applied onto) such as paper, leather, and textiles. It has been estimated that wastewater containing textile dyes contributes to ~18% of industrial water pollution [51] and that its presence in the environment has several negative impacts on aquatic ecosystems. Dyes are toxic and undesired pollutants in aquatic ecosystems because they are visible to aquatic life by interrupting photosynthetic activity even at trace concentrations. Dye molecules typically exist in complex forms (after binding with other molecules). They are generally resistant to aerobic digestion, light, heat, or oxidants, making them difficult to treat using the conventional wastewater treatment process [52,53]. Because of their complex structures and the presence of benzene rings (aromatic), they are difficult to biodegrade, and thus it is essential to physically remove them. Membrane separation is one of the premium methods used to treat wastewater laden with dyes because it is economical and effective in terms of dye retention. To increase the dye retention capacity of membranes, MMMs incorporating adsorbent filler materials have attracted attention over the years (Table 2). Some MMMs are known to enable a strong binding of the filler material with the bulk membrane polymer, thereby exhibiting good mechanical and thermal stability [54].

**Figure 4:**
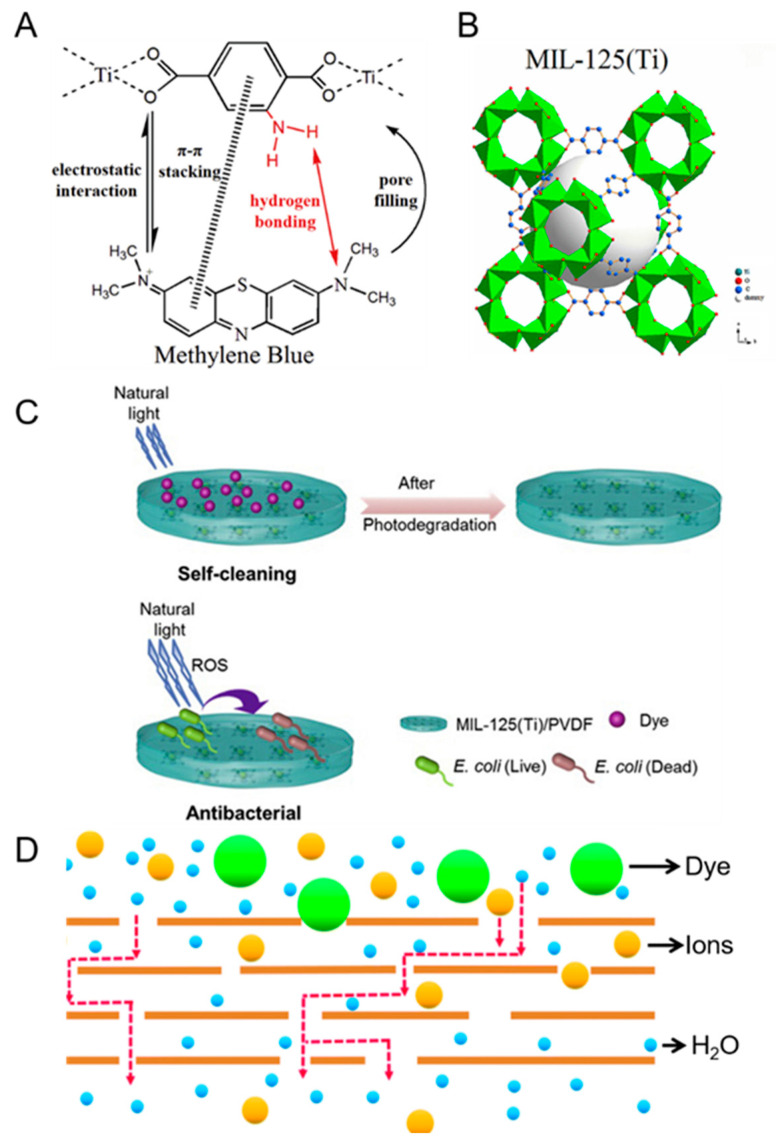
(**A**) Schematic illustration of the three possible mechanisms of methylene blue due to adsorption on Ti-MOFs: electrostatic interaction, π-π stacking interactions between the adjacent benzene rings, and hydrogen bonding [58]. (**B**) The structure of MOF MIL-125(Ti) [58]. (**C**) The illustration of the self-cleaning and anti-bacterial properties of a PVDF MMM incorporated with MIL-125(Ti) filler materials [54]. (**D**) Transport pathways of molecules with various hydrated sizes through graphene oxide sheets, modified from [59]. All figures are reprinted with copyright permissions from the respective references.

Overall, the various methods to remove dyes in water bodies using MMMs (Table 2) can be classified into three main mechanisms:

- ***Adsorption of dye molecules.*** A common approach to increase the dye rejection of membranes is to incorporate adsorbent filler materials into the membrane matrix (Table 2). For example, it was reported that the incorporation of adsorbent filler materials (e.g., GO [55], MIL-125 [54], MOF-2(Cd) [53], and SnO_2_ [56]) into the porous membrane matrices led to dye rejection improvements. For GO, it is postulated that its conjugated two-dimensional structure [55] encourages π-π stacking interactions with the dyes, thereby stimulating the adsorption of dye molecules onto the MMM. Using the same concept, empirical evidence suggests that the interaction between the electrons in the benzene ring of the dye molecule and the benzene ring in the MIL-125 (Figure 4A) can result in higher sorption rates of dye molecules onto the membrane [54]. Thermodynamic studies suggest that the adsorption of dye molecules is a spontaneous and exothermic process. The adsorption process is typical of a Langmuir isotherm whereby dye molecules adsorb onto the surface to form monolayer deposition [49,57]. In all adsorption experiments, the saturation of adsorption sites will necessitate a desorption step to regenerate the former. To facilitate the desorption of dye molecules from the adsorption sites, the typical approach is to immerse the membrane in HCl solution [49,57] or organic solvents (e.g., acetone) [58], and thereafter the membrane must be washed with deionized water to remove any leftover solutions prior to the filtration process. However, it must be noted that the adsorption efficiency of the membrane will decrease by 3–25% after 5−10 cycles of adsorption-desorption experiments because a small number of adsorption sites cannot be regenerated using facile desorption methods [49,57]. Our key message here is that a suitable adsorbent is not only one that has high adsorption capacity but also high regeneration efficiency. The latter is crucial for real-world industrial applications whereby the membrane can retain its high adsorption capacity in the long run after multiple cycles of utilization and regeneration (i.e., adsorption and desorption steps, respectively).

Besides increasing dye rejection, MMMs are also known to have improved properties with respect to the control membrane, such as improved water permeability, surface wettability, mechanical strength (e.g., reinforcing effect by GO), and decreased fouling propensity (organic and biofouling), all of which is attributed to the presence of filler material [54,55]. It is also worth noting that the dye adsorption rate is dependent on the feed pH and temperature. For example, it was reported that Congo red dye was more readily rejected (99.5%) at low pH because cations were more preferentially adsorbed onto the filler material (L. Camara) due to electrostatic attraction effects [52]. However, another study reported that the competitive sorption between dye molecules and H^+^ ions at low pH could result in lower dye adsorption rates [57]. The key message here is that there can be conflicting mechanisms under identical conditions because individual dyes have different responses and properties. Hence, the operating conditions (e.g., pH/temperature) need to be optimized with respect to the individual dye properties to maximize the desired dye removal rate. Nevertheless, it is generally true that the removal efficiency of the MMM will decrease at higher dye concentrations because of the saturation of the adsorption sites (which can lead to enhanced concentration polarization). The driving force for mass transfer across the membrane will inevitably be weaker, and water flux will decrease [52]. The key point here is that the removal efficiency of MMM is typically lower for more concentrated feeds. Hence there is a need to examine the feed characteristics before determining the feasibility of using a particular MMM for dye treatment.

- ***Photo-degradation of dye molecules.*** It was reported that MMMs containing filler material MIL-125 (Ti) (Figure 4B) were able to degrade RhB dye under natural light due to the photo-degradation effect induced by the MOF in the membrane (Figure 4C). First, the RhB dye molecules are physically adsorbed onto MIL-125 (Ti) via electrostatic attractions during the filtration process [58]. Under the presence of natural light, the sensitization of the MIL-125 (Ti) could promote electron transfer, such that electron holes (h^+^) and superoxide free radicals are produced [54]. The produced radicals are known to degrade the dye molecules and also impart antibacterial properties (Figure 4C) to the MMM because of its ability to degrade organic contaminants via photo-catalytic means (e.g., oxidizing species such as *•OH* radicals are known to destroy the cell walls of bacteria).
- ***Donnan exclusion and size effects.*** The optimization of membrane surface charge or pore size is another facile way to increase the dye rejection of porous membranes. For example, the incorporation of filler materials to render the membrane surface more negatively charged has shown success in increasing dye rejection (for negatively charged dyes such as acid black) because of electrostatic repulsion effects [46,53,55]. Secondly, dyes can be rejected via the size exclusion effect when the size of the hydrated dye ions exceeds the pore size of the membrane (Figure 4D). Typically, a combination of charge and size exclusion effects work in tandem to reject dyes [52,53]. The size exclusion effect is particularly pronounced when the dye molecules agglomerate in aqueous solutions [52]. However, for dye molecules that are of low molecular weights (such that it is in the range of the molecular weight cut-off (MWCO) of UF membranes, e.g., 618 g/mol for reactive orange-16 dye), the Donnan exclusion effect will be more dominant than the size effect [56]. Hence it is crucial to tailor the surface charge of the porous membranes based on the intrinsic charge of the dyes in a hydrated state.

### 3.3. Humic Acid and Organic Compound Removal

Polar organic compounds typically partition into the aqueous phase, thereby exhibiting high mobility in water bodies and within the hydrological cycle. Recent evidence has suggested that trace concentrations of dissolved organic pollutants in water can disrupt the food chain in aquatic ecosystems and the biological functions of living organisms [60]. One of such examples is polycyclic aromatic hydrocarbons (PAHs), a class of organic compounds produced in petroleum refining and the combustion of fuels. PAHs are known to be toxic, carcinogenic, and highly resistant to degradation via biological means [34]. Hence, these pollutants need to be physically removed, else their persistence in the environment will pose severe threats to the healthy development of aquatic ecosystems. MMMs incorporated with adsorbent filler materials have been studied as an option to increase the retention of humic acid and organic compounds from aqueous media such as brackish water or wastewater (Table 3). In general, the removal methods can be classified into the categories of dense and porous membranes.

**Figure 5:**
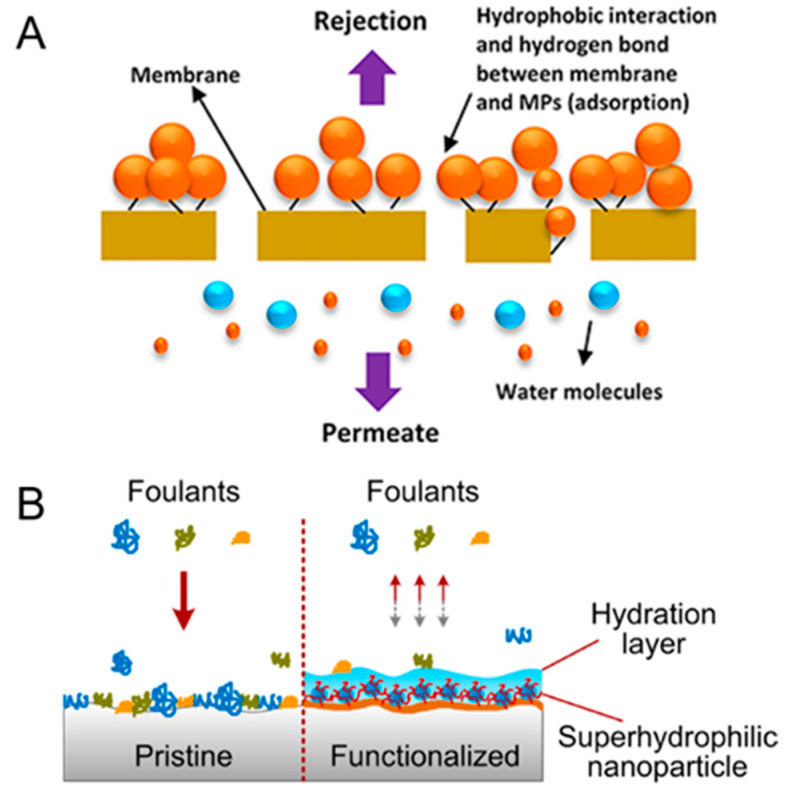
(**A**) Schematic illustration of the removal mechanism of micropollutants in porous membranes [62]. The micropollutants are preferentially adsorbed onto the membrane because of hydrophobic interaction with the membrane surface, whereas water molecules readily permeate through the membrane pores. (**B**) Schematic illustration of foulant adsorption on a pristine membrane and a functionalized membrane with a hydration layer on the membrane surface [63]. The hydration layer is formed due to the presence of hydrophilic filler materials. The hydration layer inhibits foulant adsorption, thereby reducing the propensity of membrane fouling. All figures are reprinted with copyright permissions from the respective references.

- ***Sieving and electrostatic repulsion effects in dense membranes.*** The transport phenomena in the dense selective layer of reverse osmosis (RO) membranes are well described by the solution-diffusion mechanism whereby water and solutes dissolve onto the membrane surface and thereafter diffuse through a selective layer. To increase the rejection of organic compounds in dense membranes, a potential approach is to introduce filler materials to modify the charge and pore properties of the polyamide layer. Albergamo et al. explored the use of MMM-RO membranes incorporated with zeolites and aquaporins to treat brackish water loaded with 30 persistent organic micropollutants (e.g., paracetamol and diuron) [60]. It was reported that both types of high-flux RO membranes had similar organic pollutant (OP) removal rates as compared to the TFC membranes despite having a higher solute permeability (because a higher water flux consequentially results in a higher solute flux [23]). It was reported that the MMM was more effective in rejecting neutral pollutants of molecular weight lesser than 150 Da (e.g., 1H-benzotriazole), but remained comparable to that of the TFC membrane for OPs with higher molecular weight [60]. This suggests that the molecular sieve-like nature of the filler material (e.g., a pore size of 0.3–0.8 nm for zeolites [26]) might have restricted the passage of Ops based on the size exclusion effect. In addition, anionic OPs (e.g., acesulfame) were more preferentially removed by both MMM-RO membranes because of electrostatic repulsion with the negatively charged surface of the membrane.
- ***Increase hydrophilicity and adsorption capacity in porous membranes.*** To increase the retention of OPs in porous membranes (Figure 5A), the typical approach is to modify the bulk membrane matrix properties or to incorporate adsorbents into the membrane matrix [34,43]. For example, the incorporation of MCM-41-NH_2_ filler material in UF membranes has shown success in achieving higher removal rates of polycyclic aromatic hydrocarbons (PAHs) [34]. Being a mesoporous material, MCM-41-NH_2_ fits the bill of an adsorbent material because of its uniform distribution of mesopores (pore diameter of 3.58 nm), high surface area (350 m^2^/g), and most importantly, it is mechanically stable [34]. The mechanism for the removal of PAHs by the MMM is physisorption, whereby PAH molecules are physically adsorbed onto the pore cavities of MCM-41-NH_2_. To maximize the adsorption capacity of the MMM, it is crucial to disperse the filler materials in the UF membrane matrix to ensure all the pore cavities are available for physisorption.

Dual-function MMMs have also been explored, whereby two filler materials are incorporated into the UF membrane matrix to harness the synergistic advantages of both fillers. For example, Anjum et al. reported the success of dual-function UF-MMMs incorporated with UiO-66 and Zeolite 4A for flux enhancement (~2 times higher) at a fairly similar humic acid rejection when compared against the control membrane [24]. This work highlighted that the incompatibility between filler materials could lead to agglomeration. It was reported that the optimal concentration was 1 wt.% (when the rejection of humic acid reached 99%) and that the agglomeration of filler materials occurred once the filler load exceeds 2 wt.% [24]. The incorporation of UiO-66 and zeolite 4A is known to increase the surface hydrophilicity of the UF membrane [24,39] because the presence of more hydroxyl functional groups encouraged the formation of hydrogen bonds with water molecules. This results in the formation of a hydration layer on the membrane surface (Figure 5B), rendering the adsorption of humic acid on the membrane surface more difficult and thus also resulted in a lower degree of membrane fouling.

### 3.4. Nitrates and Ammonia Removal

Ammonia nitrogen and nitrates are present in the environment due to the excretion of fish waste in water bodies. In aquatic chemistry, both the molecular and ionic forms of ammonia nitrogen exist in the form of NH_3_ and NH_4_^+^, respectively [61]. The molecular or unionized form (NH_3_) is toxic to aquatic ecosystems, and its concentration is typically low (<0.05 mg/L) due to the natural replacement of water with freshwater, as well as the biological conversion of ammonia to nitrate in biological processes (e.g., bacterial digestion). However, this process is slow due to the long start-up time and is not effective when the concentration of ammonia nitrogen is high. The excessive presence of nitrate in water bodies will also alter ecological balances due to the growth of algae which consequently inhibits the healthy growth of aquatic plants and animals. To reduce the concentration of ammonia in water bodies, various methods can be used, such as stripping using hydrophobic membrane contactors or nitrification-denitrification process using micro-organisms [35]. However, these processes are costly due to the need for various equipment and chemicals, which is not favorable from the perspective of environmental footprint. 

Membrane processes using porous UF membranes have been explored to remove ammonia and nitrates in aqueous systems (Table 3). In recent years, MMM-UF membranes have slowly gained attention due to their high efficiency to remove trace amounts of ammonia in feed water (e.g., typically <10 ppm) [61]. MMMs incorporating zeolites [35,61] and hematite (α-Fe_2_O_3_) [20] nanoparticles have been effective in removing ammonia due to the capture of ammonium or nitrate ions by the filler material embedded in the membrane. For example, NH_4_^+^ and NO_3_^−^ ions can be readily adsorbed onto zeolite and hematite particles, respectively, because of electrostatic attractions [20,35]. However, the presence of other competitive cations (such as Na^+^, Mg^2+^, which are commonly found in wastewater) will affect the adsorption capacity of the MMM for that the former can compete with NH_4_^+^ for the adsorption sites [35]. Different ions have different interactions with zeolites because of size and charge effects which dictate the sorption mechanism onto zeolites. Hence, more research on the development of materials with a more selective capture of target ions [64] is needed to enable a more precise capture of desired ions (i.e., the capture of the desired ion should be maximized while other competing ions should readily permeate through the membrane). 

Empirical evidence suggests that the adsorption capacity of the MMM is dictated by the thermodynamic equilibrium process whereby the MMM’s maximum adsorption capacity is dictated by the amount of ions adsorbed as well as the amount of ions in the feed water [35]. The regression of experimental data has also shown that the adsorption isotherm fits well to a Langmuir model, whereas the kinetics is postulated to obey the pseudo-second-order model [20]. In a typical adsorption process, rapid adsorption occurs initially because of the abundance of unoccupied adsorption sites. As the adsorption sites become saturated, the MMM’s adsorption capacity is reached, which is typically manifested by a drop in ammonia and nitrate removal efficiency. Lastly, the long-term performance of the MMMs will hinge on the ease of regeneration of adsorption sites as well as the stability of the incorporated filler materials [20,35]. The former typically requires a desorption process to recover the membrane’s adsorption capacity as well as flux, whereas the latter requires the filler materials to be securely anchored onto the polymer matrix after continuous filtration. At the current stage, the MMMs’ regeneration ability was only tested under relatively short times (typically less than 3–4 days) using a very limited range of filler materials [20,35]. It is recommended for future research to investigate the regeneration ability of MMM’s at longer timescales as well as study the stability of other adsorbent filler materials.

## 4. Perspectives and Future Outlook

### 4.1. Improvement of Removal Efficiency via Process Optimization and Combination

- ***Process optimization to improve removal efficiency.*** One of the primary concerns of membranologists developing novel membranes and membrane processes is how to balance the trade-off between membrane permeability and selectivity [26,65]. To manage the trade-off, researchers oriented more toward materials science have made much effort to discover new materials, combinations of the existing materials or optimal conditions to develop high-performance membranes with high permeability and high selectivity. However, only a single-stage module equipped with a high-performance membrane with excellent permselectivity is not enough to guarantee the high quality of processed water. Accordingly, processing variables should be considered and optimized holistically to guarantee the quality of the final product. Particularly, since MMMs remove contaminants based on size exclusion and adsorption, the contact time can affect the removal efficiency just like any other nanocomposite adsorbents [66,67,68]. Thus, our key message here is that there is a need to balance the water flux and retention time to improve removal efficiency while taking account of the optimal number of modules in series for further improvement.
- ***Process combination to improve removal efficiency.*** Pollutant removal processes using MMMs can be combined with other membrane processes for further improvement of removal efficiency. An RO process is a typical example that can be used in series to post-treat the effluent produced from UF using MMMs. An RO membrane consists of a polyamide active layer that can separate organic and ionic species by providing much dense and charged channels. However, the much dense and charged active layer of RO membranes could be a double-edged sword in that it can lead to the improved removal efficiency of the final product at the expense of lower overall productivity (in terms of water permeance) because of the permselectivity trade-off. Fortunately, the trade-off can be partially relieved by support modification [69,70,71], which is a way to enhance water permeance without compromising salt rejection by maximizing the surface porosity of a support membrane and thereby shortening the diffusion pathway across an active layer.

**Figure 6:**
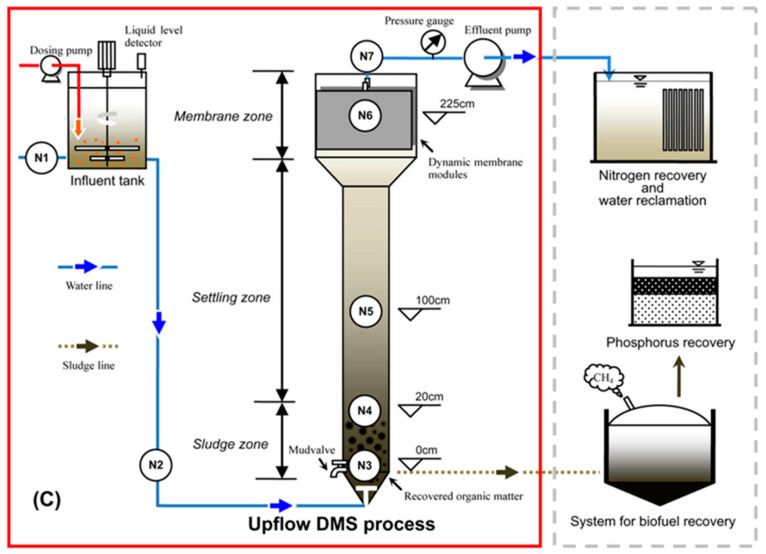
Schematic illustration of the upflow DMS process [72] (reprinted with copyright permission).

A dynamic membrane separation (DMS) process (Figure 6) could be considered as another membrane process for the post-treatment of effluents from UF using MMMs. A DMS process utilizes a cake layer formed on a very porous mesh consisting of screen openings ranging from tens of micrometers to a few millimeters as a secondary membrane to remove foulants [72,73,74]. The cake layer can be used to additionally adsorb and screen residual pollutants after UF using MMMs. Moreover, A DMS process is typically operated at a very low constant flux (e.g., 1–2 LMH [75]), such that it can elongate the hydraulic retention time (HRT) compared to conventional wastewater treatment. The longer HRT could allow dynamic membranes to spend enough time to adsorb residual pollutants, thereby increasing the quality of the final effluent. Lastly, since a DMS process can be employed in the form of gravity-driven filtration [73], the energy load caused by the additional operation of a DMS process could be minimized. The low energy footprint increases the availability of a DMS process as an alternative for the post-treatment of the effluents discharged from UF using MMMs.

### 4.2. Development of MMMs Using Biomass-Converted Carbon Materials as an Environmentally Friendly Way for Pollutant Removal

Nanomaterials have been widely used for pollutant removal as additives to prepare MMMs or building blocks for laminate membranes. Between the two approaches, nanomaterials could find a sustainable and feasible way for pollutant removal through the incorporation into MMMs instead of being used for laminate membranes, as discussed earlier in Section 1 (Introduction). However, environmentally friendly materials for pollutant removal are still in high demand. In this regard, using biomass as a sustainable resource for pollutant removal in water can kill two birds with one stone by converting waste to valuable resources that can be used for water purification, eventually contributing to opening the pathway toward a resource-efficient economy. 

Diverse kinds of biomasses are listed in Table 4. Among several biomasses, agricultural biomass is one of the main contributions to the generation of biomass waste. In China as an instance, agricultural biomass (Straw: 340 million ton; Agricultural residue: 60 million ton) accounted for about 15% of the total production (2.6 billion ton) of solid biomass including forestry wood residue, manure, municipal solid waste, and organic waste in 2010 [76]. In 2012, agricultural biomass reached 900 million tons only with crop straw, and 60% of them was incinerated or discharged [77]. This trend is predicted to continue rising as shown in the fact that more than 1 billion tons of crop straw were produced in 2018 [78], which is the case for most countries [79]. Given that a substantial portion of the agricultural biomass is typically incinerated, it is reasonable to say that using biomass for pollutant removal in water could contribute to mitigating both water and air pollution.

**Figure 7:**
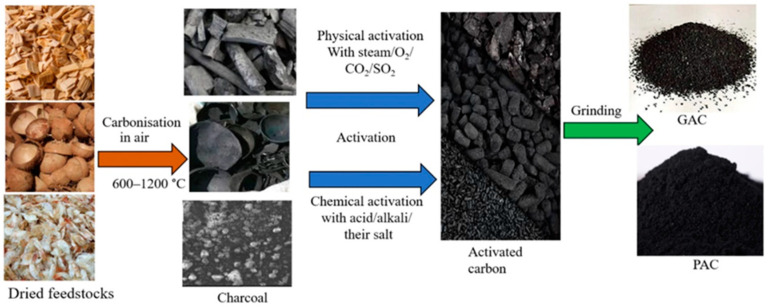
Production procedure of activated carbons from different types of biomasses [80] (reprinted with copyright permission).

Biomass can be converted into a wide variety of carbon-based materials such as biochar, carbon black, activated carbon, carbon nanotubes (CNTs), and GO via carbonization at 600–1200 °C as described in Figure 7 [80]. Carbonized materials can be treated and activated using different kinds of acids, alkalis, and gases listed in Table 4. Such activated carbon-based materials can be further modified with various kinds of materials such as biopolymers, enzymes, polymers, and organic 3D frameworks. The as-prepared carbon-based materials stemming from biomass waste can be incorporated in a membrane matrix in the same way as other kinds of carbon nanomaterials or carbon fibers [81,82] and used for pollutant removal in an aqueous phase through UF.

### 4.3. Use of MMMs for Microplastic Removal

Million tons of non-biodegradable plastic wastes have been annually discarded in the environment and weathered for the last decades, ending up with microplastics (MPs) [83]. MPs are an emerging field of study at the current stage, and more research is needed to study the environmental fate of MPs in aquatic systems. MPs are small plastic fragments (diverse sizes ranging from ~20 nm to ~5 mm) that can be found in seawater and brackish water due to anthropogenic sources such as wastewater discharged from manufacturing processes. Polyethylene plastics are a classic example of MPs used in the mass production of plastic films and healthcare commodities [84]. Although the waste streams from industries are typically treated, these MPs can easily pass through the filtration systems because of their small sizes (<1 μm), thereby ending up in water bodies when wastewater is discharged. 

**Figure 8:**
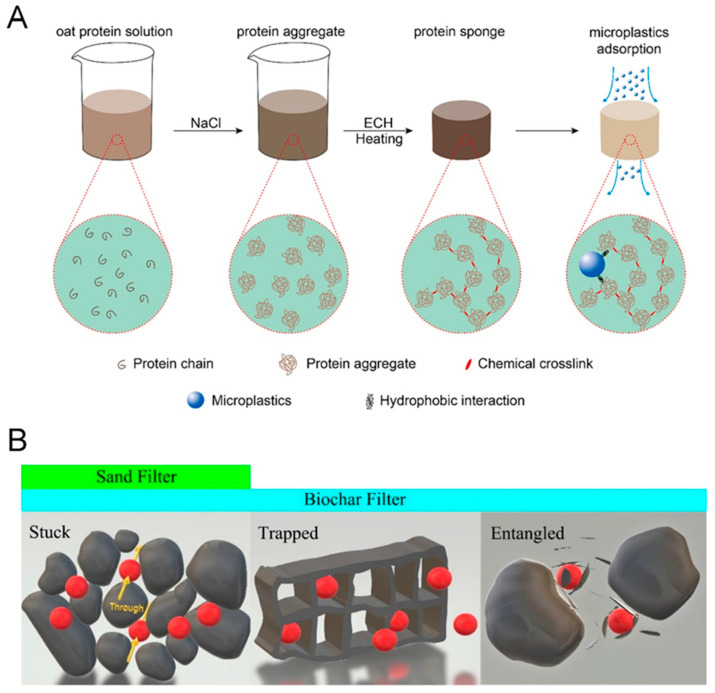
(**A**) Schematic illustration of the preparation procedure of a hydrophobic protein sponge and its application in MP removal [88]. (**B**) Three mechanisms for MP removal (i.e., Stuck, Trapped, and Entangled) [86]. All figures are reprinted with copyright permissions from the respective references.

Empirical evidence has also shown that MPs tend to degrade into smaller fragments (e.g., from micro-size to nano-size) [85], and trace amounts of organics or heavy metals can be released in the degradation process. The World Health Organization takes water pollution by MPs very seriously as an urgent global issue due to the likelihood of transport of toxic organic chemicals and heavy metals by MPs [86,87]. However, conventional sedimentation is less effective in MP removal [74]. In this respect, there is room for research on MP removal using MMMs. To the best of our knowledge, there have been no reports of MMMs dealing with MP removal. With this point in mind, we try to provide future research directions for the application of MMMs for MP removal based on the variation in their interaction behaviors depending on the size. MPs ranging from 0.02 μm to 2 μm are very similar to colloids (0.001 μm–1 μm) in size, such that the interaction between MPs and surfaces is governed mainly by the surface characteristics rather than by the pore system effects [86]. For example, a chemically crosslinked protein sponge with many hydrophobic functional groups mainly interacted with MPs via hydrophobic interaction (Figure 8A). As a result, increasing pH resulted in the protein sponge’s surface with a higher negative charge, thereby reducing MP removal efficiency by attracting more water molecules that can diminish the hydrophobic interaction between MPs and the protein sponge [88]. This result implies that MP removal efficiency could be enhanced by introducing functional groups favorable for interacting with target MPs to MMMs.

On the other hand, the potential influence of surface characteristics on the interaction between MPs and surfaces could be whittled down when MPs are larger than 10 μm. Instead, the pore system effects including morphology become predominant in the removal of MPs larger than 10 μm [86]. In detail, MP particles could be stuck by the gaps smaller than the filter particles, trapped by the pores slightly larger than MP particles, or entangled by small chips as described in Figure 8B. The removal efficiency of MPs was confirmed to be much higher when a filter had a porous structure allowing all the three removal mechanisms to be functional, which is evidenced by the comparison between biochar filters with porous structures and sand filters valid only for the ‘stuck’ mechanism [86]. However, it does not necessarily mean that we can always ignore the surface characteristics’ influence on the removal efficiency of MP larger than 10 μm. As another example, negatively charged polyethylene particles ranging from hundreds of micrometers to a few millimeters were removed with higher removal efficiency during coagulation induced by AlCl_3_·H_2_O at pH 4.0 [87]. This case shows that the charge-charge interaction between MPs and surrounding materials could still play an important role in increasing removal efficiency. From the above facts, we could conclude that it can be possible to realize the high efficiency of MP removal by incorporating adsorbents with hierarchical pore structures into a porous membrane matrix while keeping in mind that the surface property could be a significant parameter even for the removal large-size MPs. 

## 5. Conclusive Remarks

This paper covers an overview of the research progresses and trends on the emerging materials used to prepare MMMs for pollutant removal in the recent five years. For the past five years, various types of emerging materials ranging from organic to inorganic nanomaterials have been used to prepare high-performance MMMs for pollutant removal, and their effectiveness has been investigated using a range of pollutants encompassing heavy metals, dye molecules, humic acid, organic compounds, nitrates, ammonia, and so on. Most of the MMMs prepared with novel additives successfully removed more than 90% of pollutants in an aqueous phase. However, some contaminants exhibited lower removal efficiency than 50% (e.g., Ni^2+^: 44.4%; Cd^2+^: 42.8%), demonstrating a need for further improvement in removal efficiency. Moreover, high removal efficiency should be pursued, taking into account that the need for more environmentally friendly materials is growing as many countries have sought to attain a sustainable and green economy to overcome global climate change [89,90]. From the perspective of environmental sustainability, the toxic and volatile nature of some organic solvents (e.g., Dimethylformamide (DMF)) have propelled the development of bio-based polymers for membrane fabrication. For example, cellulose acetate and chitosan biopolymers were utilized to fabricate ultrafiltration membranes for water applications [50]. However, to the best of our knowledge, the development of biopolymer-based MMM for contaminant removal is still at its infancy stage, and thus more research is needed to unravel its feasibility as an alternative to synthetic polymer-based membranes. Lastly, one needs to examine whether MMMs could be used to address more complex water streams containing emerging contaminants such as microplastics, endocrine disrupting compounds, and radionuclides.

## Figures and Tables

**Table 1:** A summary of emerging materials adopted to prepare MMMs for heavy metal removal in water. The removal efficiency is reported based on the rejection of a heavy metal ion or the adsorption capacity, depending on what was reported in the literature.

Nanomaterials; Bulk Membrane Materials	Membrane Process (Operating Conditions)	Membrane Filtration Area (cm^2^)	Reported Removal Efficiency	Ref., Year
SnO_2_; Polyvinylidene fluoride (PVDF)	2 bar, dead-end filtration	29.2	Pb^2+^: 93.9%, Cu^2+^: 92.8%, Zn^2+^: 82.3%, Cd^2+^: 70.7%, Ni^2+^: 63.9%	[37], 2020
MOFs derived from amino acid S-methyl-L-cysteine; matrimid	3 bar, dead-end filtration	13.84–17.34	Pb^2+^: 98.0–98.2%	[38], 2021
UiO-66; PVDF	Filtration rates of 0.4 and 1.4 L/h, cross-flow filtration	N/A (hollow fiber)	Arsenate adsorption capacity of 267 mg/g	[39], 2020
Kaolin natural clay; polyethersulfone (PES)	0.6–1 bar, dead-end filtration	8	30% arsenic removal after 250 mins	[42], 2017
Zn:Al_2_O_3_; polysulfone (PSf)	1–5 bar, cross-flow filtration	560	Arsenic: 87% Lead: 98%	[27], 2021
α-zirconium phosphate; PVDF	1 bar, dead-end, and cross-flow filtrations	-	Pb^2+^: 91.2%, Cu^2+^: 93.1%, Zn^2+^: 44.2%, Cd^2+^: 42.8%, Ni^2+^: 44.4%	[41], 2021
Aspartic acid-functionalized graphene oxide; polyvinylchloride	2 bar, dead-end filtration	-	84% Cr rejection after 5 filtration cycles	[43], 2021
ZIF-8; PVDF	1 bar, dead-end filtration	12.56	Ni^2+^ adsorption capacity: 219.09 mg/g	[44], 2018
Zeolite; PSf	1 bar, dead-end filtration	13.4	Pb^2+^ adsorption capacity: 682 mg/g, Ni^2+^ adsorption capacity: 122 mg/g	[45], 2016
Graphene oxide (GO); PES	5 bar, dead-end filtration	3.73	Cu^2+^: ~72%, Zn^2+^: ~87%, Cd^2+^: ~68%	[46], 2020
Graphene oxide (GO); PSf	0.54 bar, cross-flow filtration	310	Cr(VI): 84%	[47], 2019
Aminated Fe_3_O_4_; chitosan/polyvinyl alcohol/PES	1 bar, cross-flow filtration	35	Cr(VI): ~85%, Pb(II): ~98%	[22], 2018
Amine modified TiO_2_; cellulose acetate	1.5 bar, dead-end filtration	-	Cr(VI): 99.6%	[48], 2018
Carboxylated cellulose fabrics	0.03 bar, cross-flow filtration	-	Adsorption capacity and rejection of Pb^2+^: 81.3 mg/g and 98.2%, respectively	[49], 2020
CuO; hydroxyethyl cellulose composite	2 bar, cross-flow filtration	33	Cr(VI): 91.4% Pb(II): 97.1%	[50], 2018

**Table 2:** A summary of emerging materials used to fabricate MMMs for enhanced dye removal.

Filler Material(s); Bulk Membrane Material	Membrane Process (Operating Conditions)	Membrane Coupon Size (cm^2^)	Reported Dye Removal/Adsorption Efficiency	Ref., Year
GO; PES	3 bar, cross-flow filtration	16	Acid black: 99.7% Rose bengal: 99%	[55], 2020
MIL-125; PVDF	4 bar, cross-flow filtration	-	RhB: 99.7%	[54], 2020
MOF-2(Cd); P84 polyimide	2 bar, cross-flow filtration	14	Methylene blue: 99.9% Eosin y: 81.2% Sunset yellow: 68.4%	[53], 2020
SnO_2_; polyphenylsulfone (PPSU)	2 bar, cross-flow filtration	N/A (hollow fiber)	Reactive black-5 (RB-5): >94% Reactive orange-16 (RO-16): >73%	[56], 2019
Lantana camara; PSf	0.5–4 bar, dead-end filtration	26	Congo red: 99%	[52], 2018
Graphene oxide (GO); PES	5 bar, dead-end filtration	3.73	Methylene blue: ~70% Methyl orange: ~88%	[46], 2020
Ca^2+^ ions; calcium alginate	0 bar, adsorption determined by immersion	-	Adsorption capacity of 3056 mg/g	[57], 2017
Carboxylated cellulose fabrics	0.03 bar, cross-flow filtration	-	Adsorption capacity and rejection of methylene blue: 77 mg/g and 98.7%, respectively	[49], 2020

**Table 3:** A summary of emerging materials used to fabricate MMMs for pollutant removal. The pollutants are categorized into two themes: (1) humic acid/organic compounds and (2) nitrates/ammonia.

Target Pollutants	Filler Material(s); Bulk Membrane Material	Membrane Process (Operating Conditions)	Reported Efficiencies/Outcomes	Ref., Year
Humic acid and organic compounds	UiO-66 and zeolite 4A; PSf	UF (2 bar, dead-end filtration)	Humic acid rejection of 99%	[24], 2020
	UiO-66; PVDF	Filtration rates of 0.4 and 1.4 L/h, cross-flow filtration	Humic acid rejection of 69–79%	[39], 2020
	Aspartic acid-functionalized graphene oxide; polyvinylchloride	2 bar, dead-end filtration	Humic acid rejection of 92%	[43], 2021
	Zeolites and aquaporins; polyamide	RO, 3–4 bar, cross-flow filtration	OP passage of AQP-RO and zeolite-RO: up 65% and 44%, respectively	[60], 2020
	MCM-41-NH_2_; PSf	11 bar, dead-end filtration	PAHs retention of 93.3–98.34%	[34], 2020
Nitrates and ammonia	Zeolites; PSf	UF (up to 3 bar, dead-end filtration)	>90% total ammonia removal	[35], 2017
	Hematite (α-Fe_2_O_3_); polyacrylonitrile	UF (1–2 bar, dead-end filtration)	Adsorption capacity of 47.7 mg/g	[20], 2018
	Zeolites; PSf	UF (1–3 bar, dead-end filtration)	95–100% ammonia removal	[61], 2018

**Table 4:** Biomass-based feedstocks, activation methods, and chemical modification methods to prepare various types of carbonaceous adsorbents. Prepared based on the information obtained from [80].

	Types	Specific Examples
Biomass-based feedstocks	Plant and crop residues	Corn cob and stalk, sorghum stalk, wheat straw, switchgrass, weeds, rice straw, rice, husk and straw, and sugarcane bagasse, etc.
	Tree and fruit residues	Wood waste, sawdust, carob, coconut husk, wheat, fruit peels, shells, and husks, etc.
	Fish and animal wastes	Crab shells, shrimp shells, leather shavings, fishery wastes, and scallops
	Marine and freshwater biomass	Microalgae, phytoplankton, and seaweeds, etc.
	Municipal organic wastes	Sewage sludge, textile sludge, paper waste, scrap tire, coffee waste, olive pumice oil, and lignin, etc.
Activation methods	Activation by gas	Steam, CO_2_, SO_2_, and O_2_, etc.
	Activation by strong alkali	NaOH, KOH, NH_4_OH, and Ca(OH)_2_, etc.
	Activation by strong acids	H_2_SO_4_, HNO_3_, and HCl, etc.
	Activation by strong acid releasing salts	CaNO_3_, ZnCl_2_, and CuCl_2_
	Activation by strong alkali liberating salts	Na_2_CO_3_ and NaHCO_3_, etc.
Materials for chemical modifications	Biopolymers	Chitosan
	Enzymes	Laccase
	Conjugated synthetic polymers	Polypyrrole and polyaniline
	Synthetic polymers	Poly(acrylic acid)
	Detergent with long hydrophobic tails	Dodecyl sulfate
	Organic 3D framework	Zeolitic imidazole

## Data Availability

No new data were created or analyzed in this study. Data sharing does not apply to this article.
